# Blood serum from individuals with Alzheimer’s disease alters microglial phagocytosis *in vitro*

**DOI:** 10.4103/NRR.NRR-D-24-01287

**Published:** 2025-06-19

**Authors:** Barbara Altendorfer, Rodolphe Poupardin, Sophie Lefèvre-Arbogast, Claudine Manach, Dorrain Y. Low, Mireia Urpi-Sarda, Cristina Andres-Lacueva, Raúl González-Domínguez, Thomas K. Felder, Julia Tevini, Marco Zattoni, Andreas Koller, Reinhold Schmidt, Paul J. Lucassen, Silvie R. Ruigrok, Chiara de Lucia, Andrea Du Preez, Catherine Helmer, Jeanne Neuffer, Cécile Proust-Lima, Aniko Korosi, Cécilia Samieri, Sandrine Thuret, Ludwig Aigner

**Affiliations:** 1 Institute of Molecular Regenerative Medicine, Paracelsus Medical University, Salzburg, Austria; 2 Experimental and Clinical Cell Therapy Institute, Paracelsus Medical University, Salzburg, Austria; 3 University of Bordeaux, Inserm, Bordeaux Population Health Research Center, Bordeaux, France; 4 Université Clermont Auvergne, INRAE, UMR1019, Human Nutrition Unit, Clermont Ferrand, France; 5 Nutrition, Food Science and Gastronomy Department, Faculty of Pharmacy and Food Science, Nutrition and Food Safety Research Institute (INSA), CIBER Fragilidad y Envejecimiento Saludable (CIBERFES), Instituto de Salud Carlos III, University of Barcelona, Barcelona, Spain; 6 Department of Laboratory Medicine, Paracelsus Medical University, Salzburg, Austria; 7 Institute of Pharmacy, Paracelsus Medical University, Salzburg, Austria; 8 Research Program for Experimental Ophthalmology and Glaucoma Research, Department of Ophthalmology and Optometry, University Hospital of the Paracelsus Medical University, Salzburg, Austria; 9 Department of Neurology, Medical University of Graz, Graz, Austria; 10 Brain Plasticity Group, Swammerdam Institute for Life Sciences, Center for Neuroscience, University of Amsterdam, Amsterdam, Netherlands; 11 École Polytechnique Fédérale de Lausanne - Brain Mind Institute - Laboratory of Behavioral Genetics, Lausanne, Switzerland; 12 Department of Basic and Clinical Neuroscience, Maurice Wohl Clinical Neuroscience Institute, Institute of Psychiatry, Psychology and Neuroscience, King’s College London, London, UK; 13 Department of Neurology, University Hospital Carl Gustav Carus, Technische Universität Dresden, Dresden, Germany; 14 Austrian Cluster of Tissue Regeneration, Vienna, Austria

**Keywords:** Alzheimer’s disease, blood serum, eicosapentaenoic acid, *in vitro* parabiosis, metabolome, microglia, omega-3 fatty acids, phagocytosis

## Abstract

In Alzheimer’s disease, microglial phagocytosis is engaged in the pathogenesis as it clears abnormal protein accumulations, debris, and apoptotic cells in the early stages of Alzheimer’s disease, but fuels neuroinflammation and accelerates disease progression in later stages. *In vivo* parabiosis experiments in aged animals have demonstrated that blood-born factors modulate synaptic plasticity, neurogenesis, and microglial responses. We hypothesize that peripheral factors can modulate microglial function and thereby possibly influence Alzheimer’s disease pathology. The objective of this study is to investigate the effects of Alzheimer’s disease serum on microglial phagocytosis. Here, we use an immortalized human microglial cell line in an *in vitro* parabiosis assay to investigate the impact of the serum from individuals diagnosed with Alzheimer’s disease (*n* = 30) and age-matched controls (*n* = 30) (PRODEM study) on microglial phagocytosis. Exposure to Alzheimer’s disease serum increased microglial phagocytic uptake of pH-sensitive fluorescent particles and downregulated expression of the lysosomal master regulator transcription factor EB (*TFEB*) and of ATPase H^+^ transporting lysosomal V1 subunit B2 (*ATP6V1B2*), a component of the vacuolar ATPase. To identify serum components that may relate to changes in phagocytosis, serum samples of the Three-City Study (3C Study) were used. In the 3C Study, blood samples were collected up to 12 years before the onset of cognitive decline or dementia and their serum metabolome is well-defined. Microglia exposed to the serum of future Alzheimer’s disease patients from the 3C Study displayed an increased phagocytic uptake compared with the serum of matched controls, depending on the presence of the apolipoprotein E ε4 allele in the Alzheimer’s disease patients. Furthermore, microglial phagocytosis correlated inversely with serum levels of the omega-3 fatty acid eicosapentaenoic acid. We confirmed this inverse correlation between eicosapentaenoic acid and phagocytosis in the serum samples of the PRODEM cohort. In addition, *in vitro* testing of eicosapentaenoic acid on microglial phagocytosis showed a concentration-dependent decrease in phagocytic uptake. In conclusion, following incubation with Alzheimer’s disease blood serum, we observed increased microglial phagocytic uptake and the downregulation of *TFEB* and *ATP6V1B2*, possibly indicating lysosomal dysfunction. Furthermore, microglial phagocytosis was inversely correlated with serum eicosapentaenoic acid levels, suggesting an important role for dietary eicosapentaenoic acid in microglial function.

## Graphical abstract

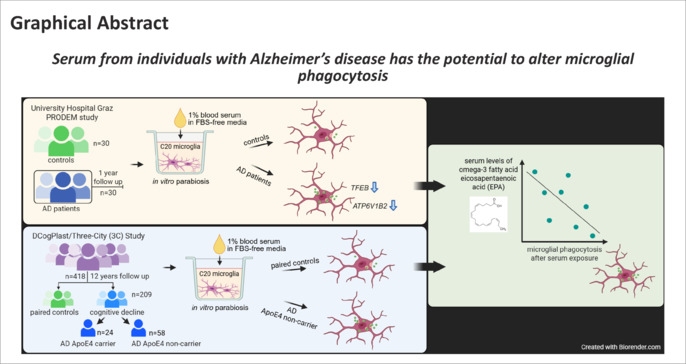

## Introduction

Microglial cells are the resident innate immune cells and professional phagocytes of the central nervous system. They play a crucial role in brain homeostasis, such as the release of neurotrophic factors, synapse pruning, and host-defense against non-self and altered-self harmful agents (Sierra et al., 2024). In age-related dementias such as Alzheimer’s disease (AD), microglia are mediators of neuroinflammation and critical players in disease progression (Mosher and Wyss-Coray, 2014; Scheltens et al., 2021). Genome-wide association studies have identified a significant number of AD risk genes that are highly or even exclusively expressed in microglia (Efthymiou and Goate, 2017), several of which are involved in phagocytosis (Podleśny-Drabiniok et al., 2020). Phagocytosis represents a key function of microglia and comprises the recognition, engulfment, and degradation of pathogens or other harmful material. With age, and particularly in neurodegenerative diseases, including AD, the essential process of phagocytosis, carried out mainly by microglia in the brain, is disturbed (Thomas et al., 2022). On one hand, clearance of debris in the brain becomes impaired, while, on the other hand, excessive phagocytosis can damage synapses, leading to neuronal dysfunction and degeneration (Galloway et al., 2019). This makes microglia a prime target for AD research.

In the context of aging, studies have highlighted the significant impact of peripheral factors, including blood serum components, on brain function (Villeda et al., 2014; Schroer et al., 2023). Parabiosis experiments, in which the blood supply of two animals is surgically connected, have shown that factors circulating in the blood of old mice have negative effects on neural plasticity and cognition (Villeda et al., 2011; Pálovics et al., 2022). Moreover, injections of plasma from old mice into young mice increased the phagocytic activity of microglia in the brain of young mice (Yousef et al., 2019). In contrast, blood plasma from young animals was able to restore neuronal function and cognitive behavior in preclinical models of AD (Middeldorp et al., 2016; Zhao et al., 2020). Recently, AD blood has been shown to affect hippocampal neurogenesis *in vitro* (Maruszak et al., 2023), but the effect on microglial function remains an open question. Changes in the blood of AD patients, such as altered levels of cytokines and metabolic factors, could influence microglial performance and thus the course of the disease.

In this study, we investigated the effects of blood serum from individuals diagnosed with AD and corresponding age-matched controls on phagocytosis and gene expression of inflammatory and lysosomal markers of human microglia *in vitro*. In addition, we use well-characterized serum samples from a cohort of older individuals, who developed cognitive decline over time, and matched-pair control samples to correlate microglial phagocytosis with their serum metabolome and to gain insight into which components of the blood may influence microglial function.

## Methods

### Cohort description

#### PRODEM study

Serum samples from individuals with dementia and clinically diagnosed with probable AD (*n* = 30, hereafter referred to as AD patients) and dementia-free controls (*n* = 30) were kindly provided by the University Hospital Graz, Graz, Austria. Collection and further use of these samples were ethically approved March 13, 2008 as part of the PRODEM study by the ethics committees of the Medical University of Graz (Protocol code: 19-135 ex 07/08) (Seiler et al., 2012; Kern et al., 2022). All participants gave written informed consent. Demographically, the AD samples consist of 19 females and 11 males with an average age of 68 years, evenly distributed over a range between 51 and 86 years with age-matched controls (**Table 1**). The cognitive performance of all the participants was assessed via the mini-mental-state examination test (MMSE) at baseline (BL) when the blood samples were taken. In addition, MMSE data was available for 21 of the AD patients after a follow-up (FU) of one year. Cognitive decline was calculated as the difference between MMSE at BL and FU (MMSE-BL minus MMSE-FU). Negative values (*n* = 3) were set to zero, assuming that improved MMSE at FU was due to daily fluctuations and not to actual improved cognition.

**Table 1:** Demographic data of the PRODEM cohort and DCogPlast/3C Study cohort

	Serum samples PRODEM				Serum samples DCogPlast/3C Study		
	AD (*n* = 30)	Controls (*n* = 30)	*P*-value		Cases (*n* = 209)	Controls (*n* = 209)	*P*-value
Sex female, n (%)	19 (63.3)	20 (66.7)			138 (66.0)	138 (66.0)	
Age, yr	51–86	49–86			65–85	66–86	
Age, yr, mean±SD	68±10.7	67.4±11.8	0.8552		76.0±4.5	76.0±4.2	0.614
MMSE-BL, mean±SD	19±3.5	28.1±1.4	< 0.0001		26.9±2.2	28.0±1.6	< 0.0001
MMSE-FU, mean±SD	17.2±4.6 (n = 21)	n.a.					

A two-tailed Student’s *t*-test with a 95% confidence interval was performed to compare demographical properties of the cohorts, such as age and Mini-Mental-State-Examination (MMSE) values at baseline (BL). *P*-values below 0.05 were considered significant. MMSE of a follow-up (FU) visit was only available from 21 of the Alzheimer’s disease (AD) patients in the PRODEM cohort. n.a.: Not available.

#### Three-City Study - DCogPlast subsample

Another set of serum samples (*n* = 418) was obtained from the Three-City Study (3C Study), more specifically from a case-control study of cognitive decline (DCogPlast) embedded in the Bordeaux cohort of the 3C Study (Low et al., 2019). In this study, older persons (≥ 65 years old) underwent a battery of cognitive tests and questionnaires on health and lifestyle. Participants’ characteristics are shown in **Table 1** and can be found in more detail here (González-Domínguez et al., 2021; Du Preez et al., 2022). Fasting blood samples were taken at BL, and participants completed a comprehensive cognitive battery at BL and at FU assessments every 2–3 years over 12 years. Participants were divided into cases with cognitive decline (*n* = 209) and their paired controls (matched for age, sex, and education; *n* = 209) on the basis of their slopes of cognitive change over 12 years (described in detail see Low et al., 2019). Dementia diagnosis was established by a committee of neurologists after reviewing all available information (including magnetic resonance imaging, when available) using the Diagnostic and Statistical Manual of Mental Disorders, 4^th^ Edition (Linard et al., 2020). The serum composition was characterized via untargeted and targeted metabolomics, described in detail before (Low et al., 2019; González-Domínguez et al., 2021; Lefèvre-Arbogast et al., 2021; Neuffer et al., 2022). The 3C Study protocol was approved 10/06/1999 by the Consultative Committee for the Protection of Persons participating in Biomedical Research at Kremlin-Bicêtre University Hospital (Paris, France). Written informed consent was obtained from all participants.

### Cell culture

The immortalized human microglial cell line C20 (Garcia-Mesa et al., 2017) was cultured in BrainPhys media (Cat# 05791, STEMCELL technologies, Saint Égrève, France) with 1% N_2_ supplement (Gibco, Fisher Scientific, Vienna, Austria), 1% penicillin/streptomycin (PAN-Biotech, Aidenbach, Germany), 100 µg/mL normocin (InvivoGen, Toulouse, France), 2.5 mM L-glutamine (Gibco), 1% fetal bovine serum (FBS) (Gibco) and 1 µM dexamethasone (Sigma-Aldrich, Merck KGaA, Darmstadt, Germany; freshly added with each media change). For experiments, media without dexamethasone were used. Cells were cultured in uncoated cell culture-treated T75 flasks (BioLite, Fisher Scientific, Vienna, Austria) and were passaged weekly with one media exchange in between. Before seeding for experiments, the cells were grown to a confluence of 90%–95%.

### Phagocytosis assay

C20 microglia were seeded into 96-well plates (BioLite) in a concentration of 1 × 10^4^ per well, in 100 µL media. Treatment was started the next day to allow the cells to settle down and attach firmly. Media was exchanged to FBS-free media containing a 1% dilution of the human serum sample. For testing the effects of eicosapentaenoic acid (EPA) on microglial phagocytosis, EPA (Cat# E2011, Sigma-Aldrich) or corresponding vehicle (ethanol) was added in various concentrations in FBS-containing media (400, 800, 1200, 2400 ng/mL; based on EPA levels observed in the human serum using targeted high performance liquid chromatography-tandem mass spectrometry; highest concentration of ethanol was 0.004%). After 24 hours of incubation, cells were exposed to pHrodo Green *Staphylococcus aureus* (*S. aureus*) BioParticles (Cat# P35367, Invitrogen, Fisher Scientific, Vienna, Austria) at a final concentration of 0.1 mg/mL. After 24 hours, cells were detached with 0.25% Trypsin/0.1% ethylenediaminetetraacetic acid (EDTA) and the content of fluorescent particles inside the cells, hereafter referred to as phagocytosis, was measured directly via flow cytometry (BD Accuri C6 Plus, BD Biosciences/Becton Dickinson Austria GmbH, Vienna, Austria). The gates and thresholds were adjusted on control conditions without pHrodo particles (**Additional Figure 1**). Each condition was measured in triplicate and the median fluorescent signal of the fluorescein isothiocyanate (FITC) channel was normalized to the media control (with FBS) of the respective plate to compensate for possible inter-plate variabilities.

**Figure 1:**
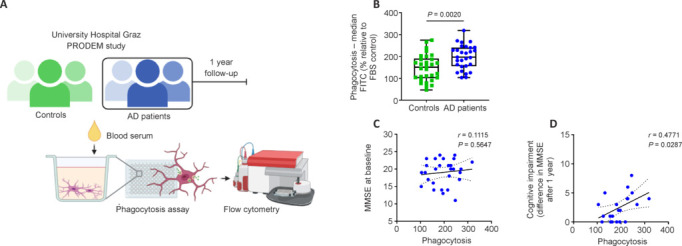
Microglial phagocytosis after human serum treatment of the PRODEM cohort. (A) Schematic representation of the *in vitro* parabiosis assay to measure microglial phagocytosis, created with BioRender.com. C20 immortalized human microglia were exposed to human blood serum of individuals diagnosed with Alheimer’s disease (AD) (*n* = 30) and age-matched controls (*n* = 30) from the PRODEM study cohort. Phagocytic uptake of pH-sensitive fluorescent bioparticles was measured via flow cytometry. (B) Microglial phagocytosis after treatment with serum from AD patients or controls. The median fluorescence of the FITC channel was normalized to the fetal bovine serum (FBS) containing media control and is expressed as a percentage. Grubb’s outlier test identified one outlier in the AD group, which was subsequently removed. A two-tailed, unpaired Student’s *t*-test was performed with a 95% confidence interval. (C) Correlation between microglial phagocytosis and the Mini Mental State Examination (MMSE) values of the AD patients at baseline (BL) (*n* = 29), which was also the date of blood collection. Pearson correlation with 95% confidence interval was performed. (D) Correlation between microglial phagocytosis and the cognitive decline of the AD patients (*n* = 21) after 1 year. Pearson correlation with a 95% confidence interval was performed. (B–D) *P*-values below 0.05 were considered statistically significant.

### Gene expression analysis

C20 microglia were seeded into 6-well plates at a concentration of 2.5 × 10^5^ per well in 2 mL of media. After 24 hours, the media was changed to FBS-free media containing 1% of human serum or FBS-containing media with 1200 ng/mL of EPA (the lowest concentration in the phagocytosis assay leading to significant effects) or the corresponding dilution of vehicle and incubated for 24 hours. Cells were washed with phosphate buffered saline (PBS) and RNA was isolated with innuPREP RNA Mini Kit 2.0 (IST Innuscreen, Ebnat-Kappel, Switzerland) following the manufacturer’s protocol for RNA extraction from eukaryotic cells. Purified RNA was eluted in 30 µL of RNAse-free water and quantified using NanoVue spectrophotometer (Biochrom, Fisher Scientific, Vienna, Austria). Reverse transcription of RNA (1 µg) to cDNA was done with GoScript Reverse Transcription System (Promega, Walldorf, Germany). The RNA was stored at –80°C and the cDNA at –20°C, until further use. Gene expression analysis using quantitative reverse transcription-polymerase chain reaction (qRT-PCR) was performed with PrimeTime qPCR Assays from Integrated DNA Technologies, Leuven, Belgium (Cluster of differentiation (CD) 68 Hs.PT.58.2488447.g, lysosomal-associated membrane protein (LAMP) 1 Hs.PT.58.27192505, LAMP2 Hs.PT.58.26112995, ATPase H^+^ transporting lysosomal V1 subunit B2 (ATP6V1B2) Hs.PT.56a.24870452, Cathepsin D (CTSD) Hs.PT.58.27568031, Microtubule Associated Protein 1 Light Chain 3 Beta (MAP1LC3B) Hs.PT.58.27295455.g, Sequestosome 1 (SQSTM1) Hs.PT.58.39829257, Beclin 1 (BECN1) Hs.PT.58.504143, Interleukin (IL) 1 Beta (IL1B) Hs.PT.58.1518186, IL6 Hs.PT.58.40226675, Translocator Protein (TSPO) Hs.PT.56a.213661, Toll Like Receptor (TLR) 2, Hs.PT.58.21312907, TLR4 Hs.PT.58.38700156.g, CD14 Hs.PT.56a.3118607.g) and TaqMan Assays from ThermoFisher Scientific (transcription factor EB (TFEB) Hs00292981_m1, Arachidonate 5-Lipoxygenase (ALOX5) Hs01095330_m1). Genes of interest were amplified with GoTaq Probe qPCR Master Mix (Promega) using 10 ng of cDNA in duplicates, on a Bio-Rad CFX96 Cycler (Bio-Rad, Vienna, Austria) using a two-step cycling protocol (95°C for 15 seconds, 60°C for 60 seconds, 40 cycles). Results are shown relative to two validated housekeeping genes (Ubiquitin C (UBC) Hs.PT.39a.22214853, Importin 8 (IPO8) Hs.PT.39a.19613208, both from Integrated DNA Technologies).

### Quantitative assessment of eicosapentaenoic acid in blood serum samples of the PRODEM cohort

Sample preparation for EPA quantification was performed according to a modified protocol described earlier (Serafim et al., 2019). For this purpose, 20 µL of EPA-d5 (10 µg/mL) as an internal standard (ISTD) and 450 µL of Hexane/2-Propanol (3:2, v/v) were added to 50 µL of serum, vortexed briefly, followed by an incubation step for 15 minutes at –20°C. Then, all samples were centrifuged at 14,000 × *g* for 5 minutes at 4°C, and the clear supernatants were dried at room temperature under nitrogen. Dried residues were dissolved in 50 µL of 2 mM ammonium acetate in 20/80 (v/v) methanol/water, vortexed for 1 minute, and centrifuged at 21,000 × *g* for 10 minutes at 4°C. The upper phases were transferred to glass vials and stored at 4°C until analysis. Chromatographic separation was carried out on an ExionLC system (Sciex, Darmstadt, Germany) using a Chromolith Performance reversed-phase column (100 × 3 mm) from Merck with a flow rate of 0.5 mL/min with 2 mM ammonium acetate in water and 2 mM ammonium acetate in 95/5 (v/v) acetonitrile/water as eluents A and B, maintained at 35°C. Gradient elution was carried out as follows: 20.0% to 95.0% B in 5.0 minutes, followed by a flushing step with 95.0% B for 3.0 minutes, followed by a re-equilibration step with 20.0% B for 1.5 minutes. The total time for a single chromatographic run was 10 minutes, and an injection volume of 5 µL was used. Selected Reaction Monitoring for EPA and the ISTD EPA-d5 in the samples was performed on a TripleQuad5500^+^ (Sciex) in negative electrospray ionization mode. Quantifier ion transitions of MS/MS detection were m/z 301.1 to 257.1 for EPA and m/z 306.0 to 262.1 for EPA-d5. Neat standard solutions were used as calibrants (0–2500 ng/mL) and QC samples (80, 400, and 2000 ng/mL). Calibration curves were derived from ratios of the peak areas of EPA and EPA-d5 to the ISTD using 1/χ-weighted linear least-squares regression of the area ratio *versus* the concentration ratio. Analyst software 1.7.1 (Sciex) was used for the acquisition of data. SciexOS (version 1.7.0, Sciex) was used for data analysis and quantification.

### Statistics

GraphPad Prism (version 9.4.0, GraphPad, Boston, MA, USA, www.graphpad.com) or R (version 4.3.1, R Core Team, 2024, https://www.R-project.org/) were used for graphical visualization and statistical analysis.

#### Statistics for the PRODEM study samples and eicosapentaenoic acid in vitro testing

An unpaired, two-tailed Student’s *t*-test was used to test differences between the two groups (controls and AD). Outliers were identified using Grubb’s outlier test. In the analysis of the microglial phagocytosis data, one outlier was identified in the AD group and subsequently removed. Pearson’s correlation was performed to analyze the correlation between cognition and microglial phagocytosis. One-way analysis of variance followed by multiple testing corrected by Tukey’s multiple comparison test was used to test the significance of different concentrations of EPA on microglial phagocytosis. For all of these analyses, a 95% confidence interval was chosen and *P*-values below 0.05 were considered as statistically significant.

#### Statistics for the 3C Study samples

All linear regression analysis for the 3C Study cohort were conducted in R (version 4.3.1, R Core Team, 2024, https://www.R-project.org/). Initial data exploration was conducted using the vis_dat and vis_miss functions from the visdat (version 0.6) package to visualize the distribution of variables and the pattern of missing data, respectively. Prior to model fitting, rows with variables with more than 50% missing values were removed to ensure sufficient statistical power. To assess the relationship between ‘Phagocytosis’ (the dependent variable) and a set of predictor variables (serum metabolites of the 3C Study samples, **Additional Table 1**), a series of robust linear regression models were fitted. Each model included ‘Phagocytosis’ and one predictor variable. The rlm function from the MASS package was used to fit the robust linear models. For each model, the *P*-value associated with the predictor variable’s coefficient (named ‘slope’ in **Table 2**), excluding the intercept, was extracted. *P*-values were adjusted using False Discovery Rate (FDR). Predictor variables with an adjusted *P*-value less than 0.05 were considered statistically significant in their association with ‘Phagocytosis’.

**Additional Table 1:** List of variables for linear regression models with microglial phagocytosis

1	1,7-Dimethyluric	47	Catechol sulfate	93	Indolelactic	139	Pregnenolone sulfate
2	1-Methylhistidine	48	Chenodeoxycholic	94	Indolepropionic	140	Proline
3	1 -Methyluric	49	Chenodeoxycholic 3 - glucuronide	95	Indoxyl sulfate	141	Proline betaine
4	1 -Methylxanthine	50	Cholesterol	96	Kynurenic	142	Propionic
5	2,6-Dihydroxybenzoic acid	51	Cholesterol sulfate	97	Kynurenine	143	Propionyl-carnitine
6	25-Hydroxyvitamin D (ng/mL)	52	Cholic	98	Lactic	144	Pyrogallol sulfate
7	2-Furoylglycine	53	Choline	99	Lauric	145	Pyroglutamic
8	2- Hydroxybenzoic acid	54	Cis-resveratrol 3 - sulfate	100	Lauroyl-carnitine	146	Pyruvic
9	2-Hydroxyphenylacetic acid	55	Citric	101	LDL	147	Retinol
10	3-(3-Hydroxyphenyl)propionic acid	56	Citrulline	102	Leucine	148	Retinol (μg/L)
11	3,4-Dihydroxybenzoic acid	57	Corticosterone	103	Linoleic	149	Serine
12	3,4-Dihydroxyphenylacetic acid sulfate	58	Cortisol	104	Linoleic acid (% of total fats)	150	Serotonin
13	3-Hydroxybenzoic acid sulfate	59	Creatine	105	Linolenic	151	Stearic
14	3-Hydroxyhippuric acid	60	Creatinine	106	Linoleoyl-carnitine	152	Stearic acid (% of total fats)
15	3-Hydroxyphenylacetic acid sulfate	61	Cyclo(L-leucyl-L-prolyl)	107	LPC(18:3)	153	Taurine
16	3 - Methylhistidine	62	Cyclo(L-prolyl-L-valyl)	108	Lutein (μg/L)	154	Testosterone
17	4,-Hydroxy-3,-methoxyphenyl-y-valerolactone sulfate	63	Cysteine	109	Lycopene (μg/L)	155	Theobromine
18	4- Hydroxyphenyllactic	64	Decanoic	110	Lysine	156	Thiamine
19	4-Hydroxyproline betaine	65	Decanoyl-carnitine	111	Margaric	157	Threonine
20	4-Methylcatechol sulfate	66	Dehydroepiandrosterone sulfate	112	Methionine	158	Total long-chain omega-3 fatty acids (EPA+DPA+DHA) (% of total fats)
21	4-Pyridoxic	67	Deoxycholic	113	Methylpyrogallol sulfate	159	Total monounsaturated fats (palmitoleic+ oleic) (% of total fats)
22	5-Hydroxyindoleacetic	68	Desmosterol	114	Myristic	160	Total omega-3 fatty acids (ALA+EPA+DPA+DHA) (% of total fats)
23	5-Hydroxytryptophan	69	Dihydroresveratrol 3-sulfate	115	Myristic acid (% of total fats)	161	Total omega-6 fatty acids (linoleic+γ-linolenic+arachidonic) (% of total fats)
24	6-Amino-5-(N-methylformylamino)-1-methyluracil	70	Docosahexaenoic	116	Myristoyl-carnitine	162	Total polyunsaturated fats (% of total fats)
25	Acetyl-carnitine	71	Docosahexaenoic acid (DHA) (% of total fats)	117	Niacinamide	163	Total saturated fats (Myristic+ Palmitic+ Stearic) (% of total fats)
26	α-Chaconine	72	Docosapentaenoic	118	N-methylpyridinium	164	Trigonelline
27	Adenine	73	Docosapentaenoic acid (DPA) (% of total fats)	119	Octanoyl-carnitine	165	Trimethylamine N-oxide
28	Alanine	74	Docosatetraenoic	120	Oleic	166	Trimethyl-lysine
29	Allantoin	75	Eicosapentaenoic	121	Oleic acid (% of total fats)	167	Tryptophan
30	Alpha linolenic acid (ALA) (% of total fats)	76	Eicosapentaenoic acid (EPA) (% of total fats)	122	Oleoyl-carnitine	168	Tyrosine
31	Arachidonic	77	Enterolactone	123	Ornithine	169	Umbelliferone sulfate
32	Arachidonic acid (AA) (% of total fats)	78	Epinephrine	124	Oxaloacetic	170	Undecanoyl-carnitine
33	Arginine	79	Ergothioneine	125	Palmitic	171	Uracil
34	Ascorbic	80	Estrone sulfate	126	Palmitic acid (% of total fats)	172	Urea
35	Asparagine	81	Gamma-linolenic acid (% of total fats)	127	Palmitoleic	173	Uric
36	Aspartic	82	Glucose	128	Palmitoleic acid (% of total fats)	174	Uridine
37	A-Tocopherol	83	Glutamic	129	palmitoyl-carnitine	175	Valeric
38	Baseline triglyceridemia	84	Glutamine	130	Pantothenic	176	Valine
39	Betaine	85	Glycine	131	Paraxanthine	177	Xanthine
40	Biotin	86	Glycochenodeoxycholic	132	p-cresol glucuronide	178	Xanthurenic
41	Blood glucose	87	Glycodeoxycholic	133	p-cresol sulfate	179	Zeaxanthin (μg/L)
42	Butyric	88	HDL	134	Pentadecanoic	180	α-Carotene (μg/L)
43	Butyryl-carnitine	89	Hippuric acid	135	Phenylacetylglutamine	181	α-Tocopherol (mg/L)
44	Caffeine	90	Histidine	136	Phenyllactic	182	β-Carotene (μg/L)
45	Carnitine	91	Hypoxanthine	137	Picolinic	183	β-Cryptoxanthin (μg/L)
46	Carnosine	92	Indoleacetic	138	Prealbumin (g/L)	184	γ-Tocopherol (mg/L)

Compounds were measured via targeted or untargeted metabolomics and are listed alphabetically. If not indicated otherwise, compounds were measured as μg/L.

**Table 2:** Association between phagocytosis and serum compounds of the DCogPlast/3C Study cohort

Compound	Slope	Adjusted *P*-value
Eicosapentaenoic acid (% of total fats)	–18.114	0.009
Total omega-3 fatty acids (ALA+EPA+DPA+DHA) (% of total fats)	–7.7	0.017
Total long-chain omega-3 fatty acids (EPA+DPA+DHA) (% of total fats)	–7.489	0.019
Carnosine (µg/L)	–0.757	0.049
Zeaxanthin (µg/L)	–0.457	0.013
Serotonin (µg/L)	–0.371	0.013
Umbelliferone sulfate (µg/L)	0.302	0.009
5-Hydroxytryptophan (µg/L)	–0.227	0.013
Lutein (µg/L)	–0.186	0.009
3,4-Dihydroxybenzoic acid (µg/L)	0.152	0.0002
Aspartic acid (µg/L)	0.148	0.012
Eicosapentaenoic acid (µg/L)	–0.009	0.025
2-Hydroxybenzoic acid (µg/L)	0.009	0.025
Threonine (µg/L)	–0.004	0.043
α-Tocopherol (µg/L)	–0.003	0.009
Citric acid (µg/L)	0.002	0.046
Palmitic acid (µg/L)	0.001	0.025
Stearic acid (µg/L)	0.0004	0.013

A series of robust linear regression models were fitted to assess the relationship between ‘phagocytosis’ (the dependent variable) and a number of predictor variables (serum metabolites of the 3C study samples, Additional Table 1). The table shows serum components significantly correlated (FDR-adjusted P-value ≤ 0.05) with microglial phagocytosis, ranked by slope (coefficient of the predictor variable) from highest to lowest (in absolute values).

## Results

### Alzheimer’s disease serum increases microglial phagocytosis and downregulates transcription factor EB

To test the impact of human AD serum on microglial phagocytic function, we used an *in vitro* model of parabiosis. Therefore, immortalized human C20 microglia (Garcia-Mesa et al., 2017) were treated with serum samples of AD patients (*n* = 30) or age-matched controls (*n* = 30) (from the PRODEM study cohort) for 24 hours and pH-sensitive fluorescent bioparticles were used to measure phagocytosis via flow cytometry (**Figure 1A**). Microglial cells showed increased phagocytic particle uptake after treatment with AD-serum in comparison to serum from controls (**Figure 1B**). To elucidate whether this increase in phagocytosis might have clinical relevance, we performed a correlation analysis with the cognitive data of the AD patients. The MMSE values of the AD patients at the beginning of the study did not correlate with microglial phagocytosis (**Figure 1C**) but interestingly, a positive association was observed with the cognitive decline occurring after 1 year (**Figure 1D**).

**Figure 2:**
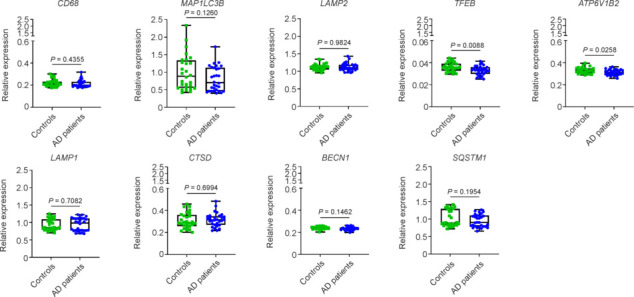
Microglial gene expression of lysosomal markers after exposure to serum of the PRODEM cohort. C20 microglia were treated for 24 hours with blood serum of Alzheimer’s disease (AD) patients (*n* = 30) and age-matched controls (*n* = 30) of the PRODEM study cohort. Gene expression of selected lysosomal markers (*CD68*, *MAP1LC3B*, *LAMP2*, *TFEB*, *ATP6V1B2*, *LAMP1*, *CTSD*, *BECN1*, and *SQSTM1*) was analyzed by quantitative reverse transcription-polymerase chain reaction. Results are shown as relative expression to housekeeping genes (Ubiquitin C - UBC and Importin 8 - IPO8). Statistical differences between AD patients and controls were assessed with a two-tailed, unpaired Student’s *t*-test with a 95% confidence interval. *P*-values below 0.05 were considered significant. ATP6V1B2: ATPase H^+^ transporting lysosomal V1 subunit B2; BECN1: Beclin 1; CD68: cluster of differentiation 68; CTSD: Cathepsin D; LAMP: lysosomal-associated membrane protein; LAMP2: lysosomal-associated membrane protein 2; MAP1LC3B: microtubule associated protein 1 light chain 3 beta; SQSTM1: sequestosome 1; TFEB: transcription factor EB.

To gain a better insight into the cellular changes following treatment with AD serum, we incubated the C20 microglia again with AD or control serum and performed gene expression analysis. Inflammation markers (*IL1B*, *IL6*, *TSPO*, and *ALOX5*) and phagocytosis receptors (*TLR2*, *TLR4*, and *CD14*) did not show differences in their transcript levels after treatment with AD serum in comparison to controls (**Additional Figure 2**). Lysosomal markers *CD68*, *MAP1LC3B* and *LAMP2* also did not differ between AD and controls (**Figure 2**). Interestingly, gene expression of *TFEB*, a master regulator of lysosomal biogenesis, was significantly downregulated upon exposure of microglia to AD serum. Further analysis of genes regulated by *TFEB*, revealed a downregulation of *ATP6V1B2*, a component of the vacuolar ATPase (V-ATPase), while the expression levels of *LAMP1*, *CTSD*, *BECN1*, and *SQSTM1* were not affected by AD serum treatment (**Figure 2**).

### Serum of future Alzheimer’s disease cases increases microglial phagocytosis depending on the apolipoprotein E ε4 allele status

The correlation between microglial phagocytosis and cognitive impairment in AD patients suggests a possible prognostic value of our *in vitro* assay, which requires a validation in another cohort. In the DCogPlast case-control study, nested in the 3C Study Bordeaux cohort (Low et al., 2019), blood samples were collected from healthy older individuals years before the onset of cognitive decline (*n* = 418). Also, their serum metabolome was characterized in detail (Low et al., 2019; González-Domínguez et al., 2021; Lefèvre-Arbogast et al., 2021; Neuffer et al., 2022). Participants’ cognitive status was assessed at BL and at FU examinations every 2–3 years for up to 12 years to assign them to either controls with preserved cognition (*n* = 209) or cases with cognitive decline (*n* = 209) (Low et al., 2019).

**Figure 3:**
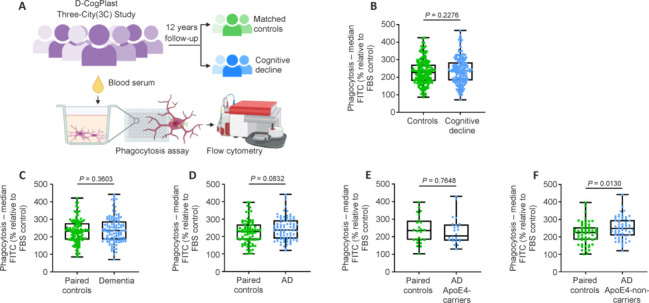
Microglial phagocytosis after human serum treatment of the 3C Study cohort. (A) Schematic representation of the *in vitro* parabiosis assay to measure microglial phagocytosis, created with BioRender.com. C20 immortalized human microglia were exposed to human blood serum from older individuals (*n* = 418) who were divided into controls and cases who developed cognitive decline after several years of follow-up (FU) (DCogPlast/3C Study Bordeaux cohort). Microglial phagocytosis of pH-sensitive fluorescent bioparticles was measured by flow cytometry. (B) Microglial phagocytosis after incubation with blood serum from controls or cases with cognitive decline (*n* = 209 per group). (C) Microglial phagocytosis after treatment with blood from individuals who developed dementia during the course of the study (*n* = 113) and their paired controls. (D) Microglial phagocytosis following exposure to blood serum from individuals diagnosed with AD during the course of the study (*n* = 82) and their paired controls. Microglial phagocytosis of AD cases were further divided into (E) ApoE4 allele carriers (*n* = 24) and (F) non-carriers (*n* = 58) and compared to their paired controls. (B–F) The median fluorescence of the FITC channel was normalized to the fetal bovine serum (FBS) containing media control and is expressed as a percentage. A two-tailed, unpaired Student’s *t*-test was performed with a 95% confidence interval and *P*-values below 0.05 were considered significant. AD: Alzheimer’s disease.

Therefore, we performed our microglial phagocytosis assay using the blood serum samples of the individuals included in the DCogPlast study (**Figure 3A**). Microglial phagocytosis did not differ between controls and cases (**Figure 3B**), even after limiting the cohort to participants who developed dementia over the years (*n* = 113) and their paired controls (**Figure 3C**). After further reduction of the cohort to individuals diagnosed with AD (*n* = 82) and their paired controls, we observed a trend (*P* = 0.0832) of higher phagocytosis in microglia treated with serum of AD diagnosed individuals (**Figure 3D**). As apolipoprotein E ε4 allele (APOE4) is a strong genetic risk factor for AD development, participants of the 3C Study were tested for their genetic profile. ApoE4 has been proposed to have opsonization functions and therefore may alter microglial phagocytosis (Shi and Holtzman, 2018). Hence, we split the group of individuals developing AD into ApoE4-carriers (*n* = 24) and non-carriers (*n* = 58) and compared the phagocytosis with the respective controls. While phagocytosis did not differ between controls and AD-ApoE4-carriers (**Figure 3E**), it was significantly increased in AD-ApoE4-non-carriers (**Figure 3F**).

### Microglial phagocytosis inversely correlates with blood levels of eicosapentaenoic acid

**Figure 4:**
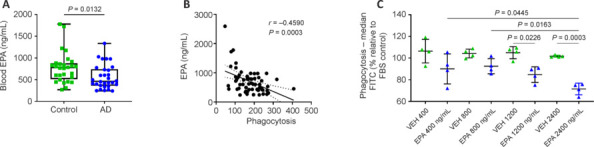
Blood levels of EPA in the PRODEM cohort and *in vitro* testing of EPA. (A) Quantification of EPA in blood serum of AD patients (*n* = 30) and controls (*n* = 30) of the PRODEM cohort, measured by mass spectrometry. A two-tailed, unpaired Student’s *t*-test was performed with a 95% confidence interval. (B) Pearson’s correlation (with a 95% confidence interval) between phagocytosis and the EPA blood serum levels of the PRODEM cohort. (C) C20 microglia were incubated with different concentrations of EPA (based on the blood serum levels) or respective vehicle control (ethanol) for 24 hours; phagocytosis was elicited with pH-sensitive fluorescent particles and measured by flow cytometry. Results are presented as the median fluorescence of the fluorescein isothiocyanate (FITC) channel normalized to the fetal bovine serum containing media control and expressed as a percentage. A one-way analysis of variance followed by multiple testing corrected by Tukey’s multiple comparison test was performed with a 95% confidence interval. For better visualization, only comparisons with a *P*-value below 0.05 are depicted. (A–C) *P*-values below 0.05 were considered statistically significant. AD: Alzheimer’s disease; EPA: eicosapentaenoic acid; VEH: vehicle.

The changes in microglial phagocytosis after exposure to AD serum raise the question of which molecular factor(s) have potentially caused this alteration. Therefore, we performed robust linear regression analysis between microglial phagocytosis and the serum metabolome data of the 3C Study samples. Variables with more than 50% missing data were excluded from analysis. From the remaining 184 parameters (**Additional Table 1**), 18 showed a significant association (*P* ≤ 0.05) with microglial phagocytosis (**Table 2**). Total omega-3 fatty acids, total long-chain omega-3 fatty acids, and especially the long-chain omega-3 fatty acid EPA showed by far the strongest correlation with microglial phagocytosis. Interestingly, EPA levels correlate significantly inversely with phagocytosis when expressed as a percentage of total fatty acids (slope = –18.114) as well as in absolute concentration (slope = –0.009). Subsequently, to validate the association of serum EPA levels with microglial phagocytosis, we measured the fatty acid EPA in the PRODEM cohort samples by targeted high performance liquid chromatography-tandem mass spectrometry. The serum concentration of EPA was significantly lower in AD patients compared with controls (**Figure 4A**), and EPA levels correlated inversely with microglial phagocytosis (**Figure 4B**), confirming the results observed in the 3C Study cohort.

### Eicosapentaenoic acid directly affects microglial phagocytosis

To decipher possible direct effects of EPA on microglial phagocytosis, we incubated the C20 microglia with different concentrations of EPA (400, 800, 1200, and 2400 ng/mL) and measured the phagocytosis after 24 hours. EPA reduced microglial phagocytosis in a concentration-dependent manner, with a statistically significant effect at 1200 and 2400 ng/mL (**Figure 4C**). This finding is consistent with the association we found in human blood serum: the higher the serum EPA concentration, the lower the microglial phagocytosis. Gene expression analysis of microglia treated with 1200 ng/mL EPA, revealed no statistically significant regulation of tested genes (*IL1B*, *IL6*, *TSPO*, *CD14*, *ALOX5*, *CD68*, *MAP1LC3B*, *LAMP2*, *TFEB*, *ATP6V1B2*, *LAMP1*, *CTSD*, *BECN1* and *SQSTM1*) compared to the vehicle control (**Additional Figure 3**).

## Discussion

Microglia are important actors in neuroinflammation and disease progression in AD. In this study, we investigated the impact of AD blood serum on microglial function using an *in vitro* parabiosis assay. Exposure to the serum of AD patients from the PRODEM cohort increased microglial phagocytic uptake, which correlated with the subsequent cognitive decline of the patients. Further, gene expression analysis revealed a downregulation of the lysosomal genes *TFEB* and *ATP6V1B2*, possibly an indication of lysosomal dysfunction upon AD serum exposure. To identify potential serum components influencing microglial phagocytosis, we used well-characterized blood serum samples from participants of the 3C Study on cognitive decline. In this cohort, microglial phagocytosis was increased in the presence of serum from individuals developing AD (ApoE4 non-carriers) and was negatively associated with the blood serum levels of the omega-3 fatty acid EPA. We confirmed this association between phagocytosis and EPA in the PRODEM cohort as well. Further *in vitro* testing confirmed concentration-dependent effects of EPA on microglial phagocytosis.

Microglial phagocytosis is an intensively studied process in the field of AD, not only in terms of amyloid-beta plaque clearance (Cai et al., 2023), but also in terms of synaptic and neuronal loss (Rajendran and Paolicelli, 2018). The ability of myeloid cells, such as microglia, to phagocytose is considered to decrease with age and in neurodegenerative diseases (Gabandé-Rodríguez et al., 2020). However, it has been observed that inflammation-triggered phagocytosis can lead to or at least contribute to neuronal cell death and neurodegeneration (Neher et al., 2011; Brown and Neher, 2012; Butler et al., 2021). Despite extensive investigations, the exact role of microglial phagocytosis in AD remains elusive. In our *in vitro* parabiosis system, we used unconditioned microglia in a non-proinflammatory state and exposed them to AD blood serum, which resulted in increased phagocytosis compared to serum of controls. While higher phagocytosis could reflect an overall pro-inflammatory state (Churchward et al., 2018), no changes were observed in the gene expression levels of *IL1B*, *IL6* and *TSPO*, three commonly used inflammatory microglial markers (Rabaneda-Lombarte et al., 2019; Bader et al., 2023).

The gene expression analysis of AD serum-treated microglia further revealed a downregulation of *TFEB* and *ATP6V1B2*. TFEB is a master regulator of lysosomal biogenesis and orchestrates a whole network of lysosomal genes (Tan et al., 2022). One element of this network is the V-ATPase, the proton pump responsible for lysosomal acidification, of which ATP6V1B2 is a subunit. Disturbances in lysosomal function, including V-ATPase, have been implicated in AD pathology (Colacurcio and Nixon, 2016; Kim et al., 2023) and a very recent study demonstrated that TFEB regulates V-ATPase function in microglial degradation of the protein tau (Wang et al., 2024). Similarly, stimulation of TFEB was found to reduce protein deposition in different mouse models of AD (Yang et al., 2022; Xie et al., 2023). We show, to our knowledge, for the first time, that serum from AD patients has the potential to negatively affect microglial lysosomal processing by influencing TFEB expression. Since the serum profiles of the PRODEM samples were not available, we can only speculate about possible factors affecting TFEB expression. Tiribuzi et al. (2014) showed a downregulation of TFEB in monocytes and lymphocytes isolated from the blood of AD patients. They associated this downregulation with increased expression of the microRNA miR-128 in blood cells, which had an inhibitory effect on TFEB. Remarkably, miR-128 has been found to be increased in the blood serum of individuals with AD compared to controls (Zhang et al., 2021).

Interestingly, we discovered a correlation between the phagocytosis of AD serum-treated microglia and cognitive decline of the patients. In terms of prognostic value, we therefore wondered whether microglial phagocytosis is also altered by the serum of individuals before the development of AD. To answer this question, we took advantage of the 3C Study cohort, where blood samples of the participants were collected up to 12 years before cognitive decline. Exposing microglia to serum of individuals who developed AD during the course of the study, we observed a trend towards increased microglial phagocytosis compared to control serum. In our *in vitro* setting, microglial phagocytosis was specifically increased when treated with serum from AD or future AD patients (not significantly), but not by serum of participants with other forms of dementia. The AD-specific increase in phagocytic activity could possibly be related to tau protein, which is found in AD blood and can serve as a biomarker to distinguish AD from other forms of dementia (Chouliaras et al., 2022). Indeed, it has been shown that extracellular tau can increase microglial phagocytosis of microspheres (Pampuscenko et al., 2020).

Interestingly, microglial phagocytosis was only significantly increased upon exposure to the serum of future AD patients who did not carry the ApoE4 allele, the strongest genetic risk factor for late-onset AD. Besides its role in lipid homeostasis, ApoE binds to apoptotic neurons and amyloid-beta plaques and modulates their phagocytosis by microglia (Atagi et al., 2015; Shi and Holtzman, 2018). This process of opsonization is suspected to be altered by expression of the E4 allele (Shi and Holtzman, 2018). Our results, in which serum from ApoE4 non-carriers induced higher microglial phagocytosis, may indicate that the opsonization capacity of ApoE4 is lower in terms of microglial phagocytosis than the more common E3 allele. Unfortunately, we do not have data on the APOE4 status of the PRODEM cohort and can thus not compare whether the differences in phagocytosis are similarly dependent on APOE4 as found in the 3C Study cohort.

Utilizing the available metabolomic data from the serum samples of the 3C Study cohort, we performed linear regression analysis that revealed an inverse association between microglial phagocytosis and the omega-3 fatty acid EPA. Omega-3 fatty acids are well known for their anti-inflammatory properties (Poggioli et al., 2023) and low plasma levels of EPA have been associated with an increased risk for dementia (Samieri et al., 2008). In addition, AD patients exhibit decreased blood levels of EPA and DHA compared to controls (de Wilde et al., 2017). In our study, AD patients of the PRODEM cohort also had lower levels of serum EPA compared to healthy controls. Furthermore, microglial phagocytosis after treatment with serum from the PRODEM study was inversely correlated with serum EPA levels, confirming the association we found in the 3C Study cohort. The decreasing microglial phagocytic uptake with increasing EPA concentration is in line with the known anti-inflammatory effects of omega-3 fatty acids. The effects of serum EPA on microglial phagocytosis was corroborated *in vitro*, where we observed that higher doses of EPA reduced microglial phagocytic uptake. However, this result is in contrast to previous studies, which observed that EPA increases phagocytosis, despite its anti-inflammatory effects, in different immune cells of human and animal studies (Hjorth and Freund-Levi, 2012) and also in a human microglial cell line (Hjorth et al., 2013). Nevertheless, it is important to highlight that phagocytosis is a biological process that strongly depends on the phagocytic target. While Hjorth et al. (2013) used amyloid beta peptides to elicit phagocytosis, our phagocytosis assay was performed with bio particles from *S. aureus*.

The use of *S. aureus* particles for phagocytosis is one of the limitations of our study. Amyloid beta or tau peptides would represent more biologically relevant phagocytic substances for AD. However, a comparison of phagocytosis of different targets is beyond the scope of this study. *S. aureus* bioparticles are a highly standardized and commercially available substrate for phagocytosis, which ensured the reproducibility of our phagocytosis assay, as we measured a large number of samples (478 in total). More importantly, the use of *S. aureus* bio particles, which are larger than a peptide, limits the uptake process to phagocytosis and minimizes other mechanisms such as pinocytosis, while it may simulate the uptake of bigger molecules such as protein aggregates (e.g., amyloid aggregations). In addition, *S. aureus* has been shown to stimulate uptake via TLR2, which is reported to bind to amyloid beta (Liu et al., 2012). Another limitation of our study is the absence of detailed information about serum immunoglobulins or complement factors, which may alter the opsonization-mediated phagocytic uptake. Further, it has to be noted that our *in vitro* parabiosis assay is an artificial system. The C20 microglial cells are an immortalized cell line and, although featuring important and specific microglial characteristics (Garcia-Mesa et al., 2017), they might not reflect the complete response these cells display in a more physiological condition. With the flow cytometry-based phagocytosis assay, we measured the content of pH-sensitive fluorescent particles inside the microglia and termed it phagocytic uptake, as we did not observe any measurable degradation of the particles in the experimental setting used (data not shown). However, we cannot completely exclude the possibility that minor effects of serum treatment on lysosomal function may have influenced the results. Furthermore, not all circulating blood factors may directly affect parenchymal microglia *in vivo*, but may act indirectly via stimulation of endothelial cells of brain vessels (Yousef et al., 2019). However, leakage of the blood-brain barrier is one of the hallmarks of AD (Nehra et al., 2022), facilitating direct contact of microglia with blood components. Notably, fatty acids such as EPA have been suggested to enter the brain not only via specialized lipid transporters, but also by simple diffusion (Mitchell and Hatch, 2011), and may therefore also directly influence microglial function.

In conclusion, our data suggest that serum of AD patients has the potential to alter microglial phagocytosis via increased particle uptake and deterioration of lysosomal function through downregulation of TFEB and a subunit of the V-ATPase. Furthermore, microglial phagocytosis was inversely correlated with blood levels of EPA, highlighting the importance of this omega-3 fatty acid in AD disease progression.

## Additional files:

***Additional Figure 1:*** *Gating strategy of the phagocytosis assay measured by flow cytometry.*

Additional Figure 1 Gating strategy of the phagocytosis assay measured by flow cytometry.
(A, C) Gate for C20 microglia was kept the same for all measurements. (B) Threshold for microglia positive for internalized bioparticles was set on a negative control where no bioparticles were added to the cells and was kept the same for all measurements. (D) Nearly all C20 microglia internalized fluorescent particles in their lysosomal compartments after 24 hours and the fluorescent peak is clearly visible right of the threshold line (gate V2-R).

***Additional Figure 2:*** *Microglial gene expression of inflammation markers after exposure to blood serum of PRODEM cohort.*

Additional Figure 2 Microglial gene expression of inflammation markers after exposure to blood serum of PRODEM cohort.
C20 microglia were exposed to 1% human serum for 24 hours before RNA isolation. Gene expression of selected markers was analyzed using quantitative reverse transcription-polymerase chain reaction. Data is shown as relative quantities compared to the two housekeeping genes UBC and IPO8. A two-tailed, unpaired student’s *t*-test was performed with a 95% confidence interval. *P*-values below 0.05 were considered as statistically significant.

***Additional Figure 3:*** *Microglial gene expression after treatment with EPA.*

Additional Figure 3 Microglial gene expression after treatment with EPA.
C20 microglia were treated with EPA or vehicle for 24 hours before RNA isolation. Gene expression of selected markers was analyzed using quantitative reverse transcription-polymerase chain reaction. Data is shown as relative quantities compared to the two housekeeping genes *UBC* and *IPO8*. An ordinary oneway analysis of variance followed by multiple testing corrected by Tukey’s multiple comparison test was performed with a 95% confidence interval. *P*-values below 0.05 were considered as statistically significant. EPA: eicosapentaenoic acid.

***Additional Table 1:*** *List of variables for linear regression models with microglial phagocytosis.*

## Data Availability

*All relevant data are within the paper and its Additional files*.
