# Supplementary figures and images for: Blood serum from individuals with Alzheimer’s disease alters microglial phagocytosis in vitro
Source: Neural Regen Res. 2025 Jun 19;21(6):2433–9. doi: 10.4103/NRR.NRR-D-24-01287 (PMC13211817; doi:10.4103/NRR.NRR-D-24-01287)

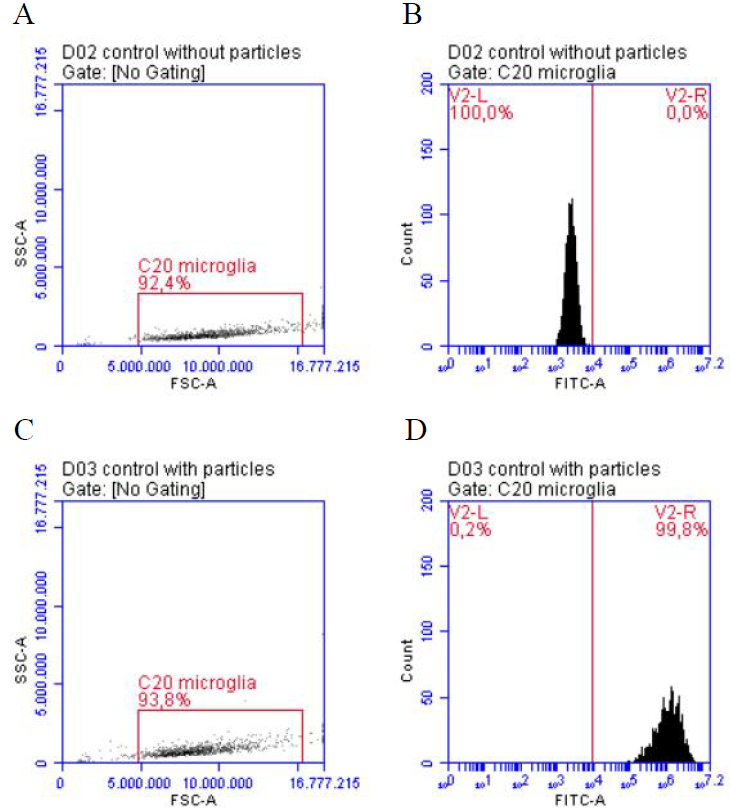

Supplement: Supplementary file 1 [file NRR-21-2433_Suppl1.tif]

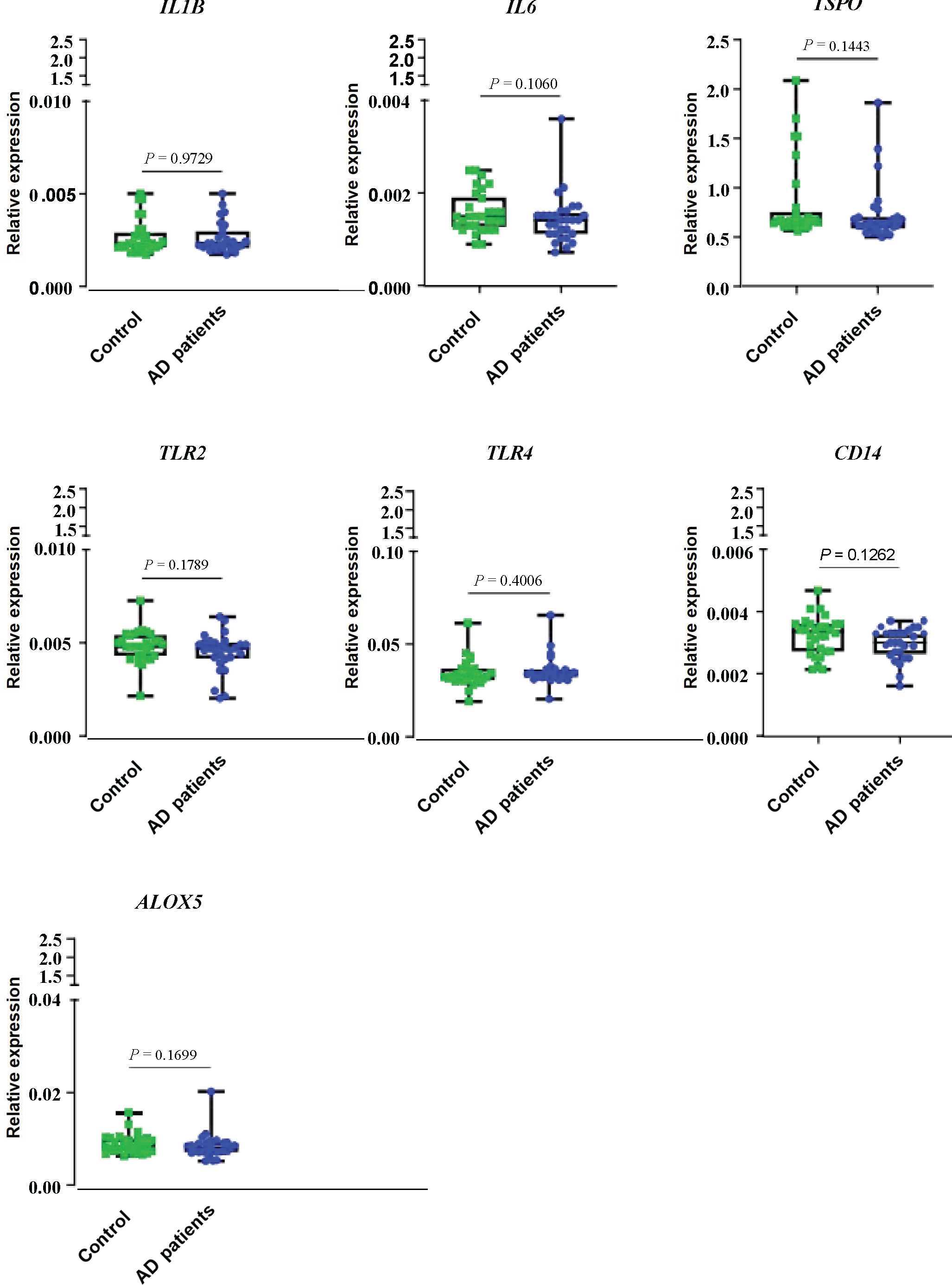

Supplement: Supplementary file 2 [file NRR-21-2433_Suppl2.tif]

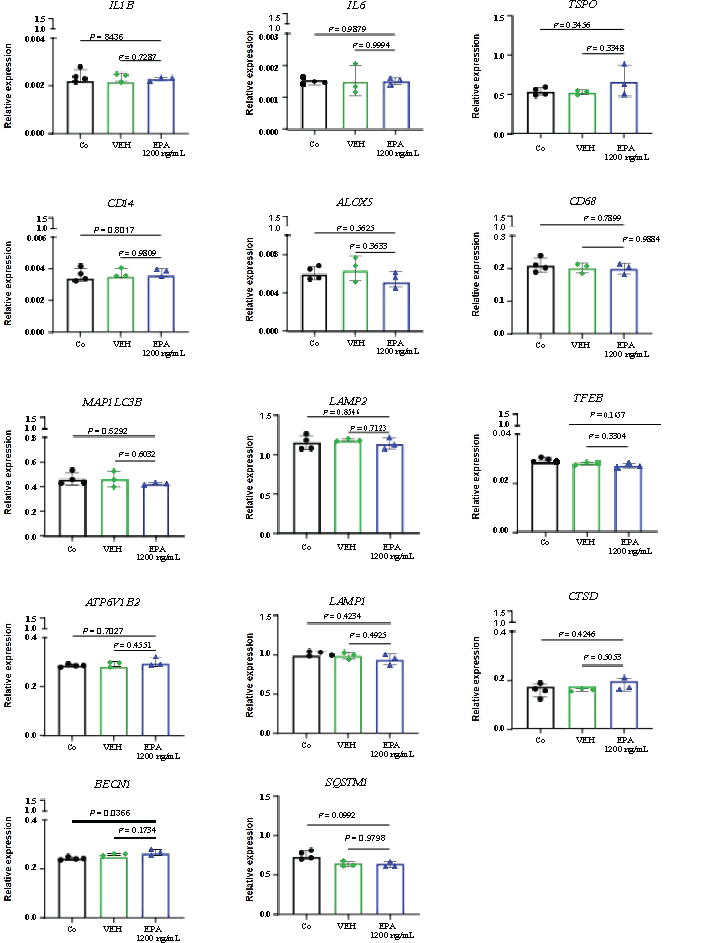

Supplement: Supplementary file 3 [file NRR-21-2433_Suppl3.tif]
